# An Assistive Role of a Machine Learning Network in Diagnosis of Middle Ear Diseases

**DOI:** 10.3390/jcm10153198

**Published:** 2021-07-21

**Authors:** Hayoung Byun, Sangjoon Yu, Jaehoon Oh, Junwon Bae, Myeong Seong Yoon, Seung Hwan Lee, Jae Ho Chung, Tae Hyun Kim

**Affiliations:** 1 Department of Otolaryngology & Head and Neck Surgery, College of Medicine, Hanyang University, Seoul 04763, Korea; hayoungbyun@hanyang.ac.kr (H.B.); shleemd@hanyang.ac.kr (S.H.L.); 2 Machine Learning Research Center for Medical Data, Hanyang University, Seoul 04763, Korea; kiddyu1991@gmail.com (S.Y.); ojjai@hanyang.ac.kr (J.O.); drbae@hanyang.ac.kr (J.B.); aodtod84@gmail.com (M.S.Y.); 3 Department of Computer Science, Hanyang University, Seoul 04763, Korea; 4 Department of Emergency Medicine, College of Medicine, Hanyang University, Seoul 04763, Korea; 5 Department of HY-KIST Bio-Convergence, College of Medicine, Hanyang University, Seoul 04763, Korea

**Keywords:** machine learning, artificial intelligence, tympanic membrane, otitis media, resident physician

## Abstract

The present study aimed to develop a machine learning network to diagnose middle ear diseases with tympanic membrane images and to identify its assistive role in the diagnostic process. The medical records of subjects who underwent ear endoscopy tests were reviewed. From these records, 2272 diagnostic tympanic membranes images were appropriately labeled as normal, otitis media with effusion (OME), chronic otitis media (COM), or cholesteatoma and were used for training. We developed the “ResNet18 + Shuffle” network and validated the model performance. Seventy-one representative cases were selected to test the final accuracy of the network and resident physicians. We asked 10 resident physicians to make diagnoses from tympanic membrane images with and without the help of the machine learning network, and the change of the diagnostic performance of resident physicians with the aid of the answers from the machine learning network was assessed. The devised network showed a highest accuracy of 97.18%. A five-fold validation showed that the network successfully diagnosed ear diseases with an accuracy greater than 93%. All resident physicians were able to diagnose middle ear diseases more accurately with the help of the machine learning network. The increase in diagnostic accuracy was up to 18% (1.4% to 18.4%). The machine learning network successfully classified middle ear diseases and was assistive to clinicians in the interpretation of tympanic membrane images.

## 1. Introduction

Artificial intelligence (AI) technologies have been introduced and applied in various medical fields. Emerging innovations regarding automatic interpretation and diagnosis from medical images have had a substantial impact on medical practice [1]. Recent investigations showed excellent results in the interpretation of various medical images including chest X-rays, mammograms, and computed tomography scans [2]. In the otolaryngology field, a deep learning algorithm successfully diagnosed maxillary sinusitis from a plain Waters’ view radiograph [3,4]. Furthermore, AI technology is being used not only to interpret medical images but also to predict the prognosis of disease. AI technology is also being expanded to determine physical examination findings and treatment plans [5,6].

Automatic diagnosis of ear diseases can be especially helpful. Despite the use of antibiotics, otitis media is a major health burden that leads to hearing loss, especially in children and the elderly [7]. Tympanic membrane evaluation is the first examination for a diagnosis of ear disease. However, this evaluation can be difficult to perform correctly in the absence of an otolaryngologist, a situation that arises frequently in emergency rooms and rural area clinics. Machine learning network assistance in the diagnosis of ear disease could expand medical accessibility for those without access to otolaryngologists by assisting nonspecialists in diagnosing otitis media. Although recent investigations with diagnostic networks for tympanic membrane images had an accuracy of approximately 90% in each setting, there are no studies concerning the educational or assistive role of machine learning networks in the diagnosis of middle ear diseases [8,9,10].

One aim of the present study was to evaluate the assistive role of a machine learning network in classifying tympanic membrane images. We designed a robust machine learning network that we applied to assist low-grade resident physicians in correctly diagnosing abnormalities on tympanic membrane images.

## 2. Materials and Methods

### 2.1. Acquisition of Tympanic Membrane Images

**Figure 1:**
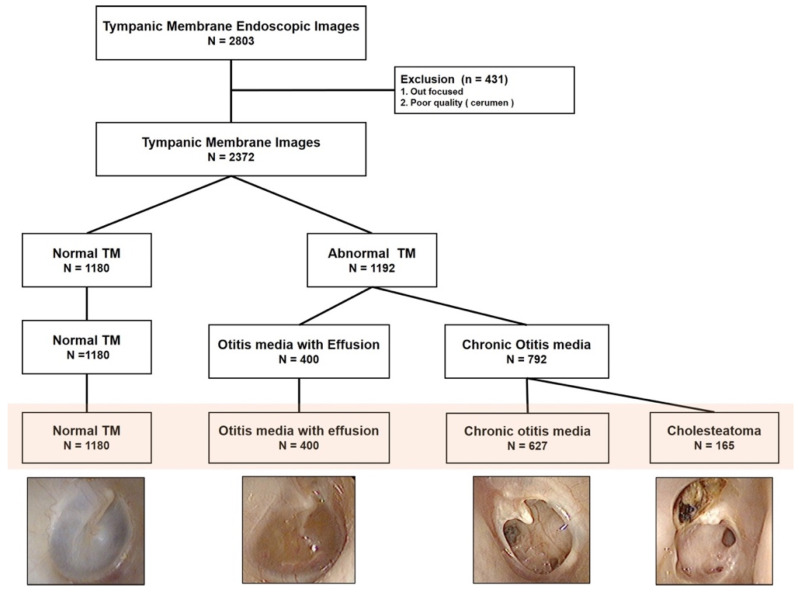
Acquisition and labeling of the tympanic membrane images.

The medical records and tympanic membrane images of patients who visited the otolaryngology department for ear problems from January 2015 to December 2018 were retrospectively reviewed. Tympanic membrane images were obtained using a Digital Videoscope (ENF-V2, Olympus) and were stored in the Picture Archiving and Communication System. Images were extracted and saved in JPEG format with a resolution of 640 × 480. Each tympanic membrane image was annotated with the correct diagnosis of normal, otitis media with effusion (OME), chronic otitis media (COM), or cholesteatoma by three otolaryngology specialists based on endoscopic images and corresponding medical records including operation records or audiologic tests. OME was diagnosed by the presence of middle ear effusion with an intact tympanic membrane. COM refers to the conditions of a perforated tympanic membrane with or without otorrhea. Cholesteatoma was diagnosed based on the presence of a retraction pocket or bony destruction with cholesteatoma debris (Figure 1). Images with other diagnoses or poor quality were excluded from the present study.

### 2.2. Data Preprocessing and Augmentation

Unrefined tympanic membrane images captured with an endoscope include unnecessary black margins on the borders, resulting in the need for a proper preprocessing step that removes these regions and thereby boosts the inference speed. Moreover, conventional data augmentation techniques are required because difficulty arises in obtaining many training datasets. Therefore, for data preprocessing, we first removed the black margins from the original image to include only the tympanic membrane and then resized the image to a 270 × 270 resolution. Next, we augmented the training images by applying random transformations (e.g., flip, flop, and rotation) and randomly cropping 256 × 256 patches from the 270 × 270 images during the training process. This served to increase the amount of training data and to secure robustness against geometric changes such as scale, translation, and rotational transformation.

### 2.3. Tympanic Membrane Diagnosis by Image Classification

**Figure 2:**
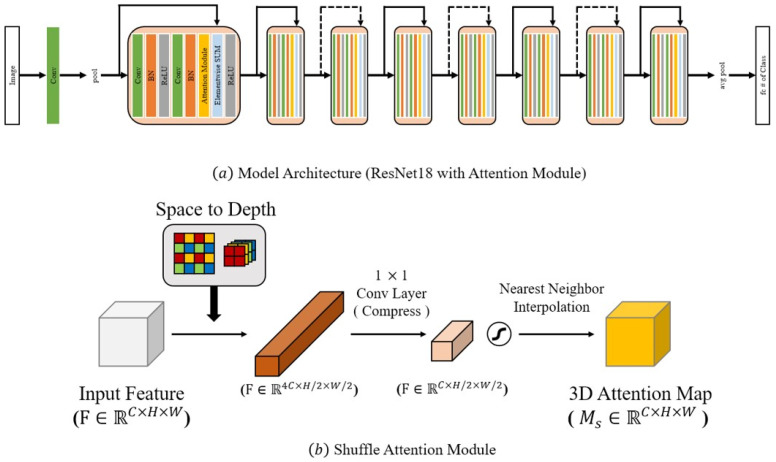
Network architecture for tympanic membrane diagnosis.

We employed conventional image classification networks for the tympanic membrane diagnosis. Specifically, our network architecture was based on a residual neural network (ResNet) [11] In particular, ResNet used residual mapping to successfully address the issue of vanishing gradients in training convolutional neural networks (CNNs) [11]. This residual mapping can be achieved using skip connection between layers to enable the network to be implemented in multiple layers. Moreover, we employed a novel attention mechanism that improved the classification performance with a few additional parameters in feed-forward CNNs. Our attention mechanism generated 3D attention maps that represented both spatial and channel dimensions through a simple operation called space to depth in the given input feature map. These attention maps refined the input feature map to increase the representation power. The overall network architecture of the proposed method is illustrated in Figure 2a.

### 2.4. Efficient Attention via the Space to Depth Approach

The attention mechanism plays an important role in image classification. Recently, the Convolutional Block Attention Module (CBAM) inferred attention maps along two separate dimensions, channel and spatial, from a given input feature map [12]. However, the spatial attention map generated by CBAM served only the same 2D spatial information to every channel dimension of the 3D input feature map [12] We presented an efficient attention module (Shuffle Attention Module) that generated the 3D attention map with a few additional parameters. Specifically, we used the space to depth operation on the intermediate feature map in the attention module; this extended the feature information around the disease area to the channel dimension so that the attention module could refine 3D feature information with simple channel-wise computing. As shown in Figure 2b, we applied the space to depth operation, which reduced the height and width of the feature by half and quadrupled the channel dimension. On this intermediate feature map, we applied channel-wise compression using 1 × 1 convolution. After the sigmoid function, we produced the 3D attention map with the same size as the input feature by increasing the resolution through nearest neighbor interpolation. Last, we multiplied the 3D attention map to the input feature map. The proposed attention module generated a 3D attention map that significantly increased the performance of the network with some additional parameters.

### 2.5. Network Performance and Validation

From 2272 tympanic membrane images, 71 representative cases were selected and used to assess the network performance. The cases consisted of difficult and demanding cases chosen by senior otolaryngologists. Several experiments to evaluate the performance of ResNets with different settings were conducted to select the best performing network. With the final network model, we conducted a K-fold (k = 5) cross validation, the standard verification technique to detect overfitting of the network. Performance verification was achieved through experiments from a total of five training sessions and was conducted by dividing the training set and validation set at 8:2 along with a fixed test set.

### 2.6. Visual Verification via Grad-CAM

We employed Gradient-weighted Class Activation Mapping (Grad-CAM) for the visual verification of the diagnostic results of the proposed method [13]. Grad-CAM produces a highlighted localization map that shows the important regions in the image for predicting middle ear diseases using the gradients of any target class flowing through the final convolutional layer in CNNs [13]. We applied Grad-CAM to the last convolutional layer placed before the fully connected layer for the visual verification of the diagnostic results of the machine learning network.

### 2.7. Assistive Role for Otitis Media Diagnosis

Seventy-one representative cases were randomly presented to low-grade (1st and 2nd) resident physicians who were asked to diagnose the associated middle ear disease of OME, COM, or cholesteatoma. After diagnosis based on the tympanic membrane images, the machine learning network diagnosis was presented. Physicians were allowed to change their final diagnosis after considering the network answer. The percentage of correct answers before and after receiving network help was calculated.

### 2.8. Ethical Issues

This investigation was approved by the Ethics Review Board of Hanyang University Guri Hospital (IRB #2019-11-016) and was performed in accordance with the Declaration of Helsinki and good clinical practice guidelines. Informed consent was waived because of the retrospective nature of the study, and the analysis used anonymous clinical data after approval of the ethics review board.

## 3. Results

### 3.1. Tympanic Membrane Images

In this study, 2272 tympanic membrane images were used. As described in Figure 1, the numbers of normal tympanic membrane cases, otitis media with effusion cases, chronic otitis media cases, and cholesteatoma cases were 1180, 400, 627, and 165, respectively. Since the prevalence of each disease was different, similar numbers of typical cases were selected to build the dataset of 71 representative cases. The representative tympanic membrane images consisted of 19 normal tympanic membrane images, 17 images each for otitis media with effusion and chronic otitis media, and 18 images for cholesteatoma and were used for final validations.

### 3.2. Selection of Best Performance Algorithm

For comparison, original ResNet (18 and 50), ResNet (18 and 50) with CBAM, and ResNet18 with our shuffle attention module were used. Table 1 shows the number of parameters in each network and the accuracy of the test set. ResNet (18 and 50) with CBAM had a lower performance in representative cases despite a slight increase in parameters compared to the original ResNet (18 and 50). In contrast, ResNet18 with the shuffle attention module required more parameters than the others but showed the best performance. Therefore, we used ResNet18 with the shuffle attention module as our final model

### 3.3. Network Verification with k-Fold Cross Validation

As shown in Table 2, the average accuracy of the validation set was 93.69% (92.4~95.6%). These results demonstrate that, despite a small amount of training data, we avoided overfitting and achieved a sufficiently robust performance in the four-class tympanic membrane diagnostic network.

### 3.4. Regions of Interest for Tympanic Membrane Diagnosis

**Figure 3:**
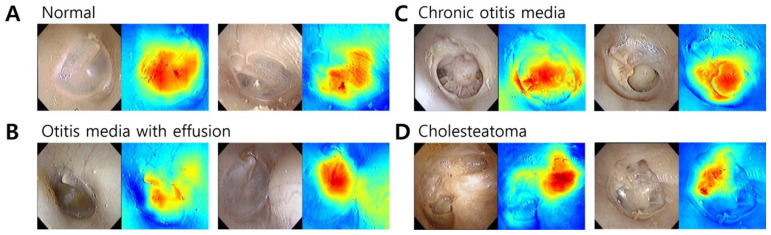
The regions of interest for tympanic membrane diagnosis were visualized as a heat map using Gradient-weighted Class Activation Mapping (Grad-CAM). (**A**) Grad-CAM focused on the pars tensa region of the normal tympanic membrane. (**B**) The change in color of the tympanic membrane due to middle ear effusion is important for the diagnosis of otitis media with effusion in the Grad-CAM analysis. (**C**) Chronic otitis media could be distinguished by perforation and remnant tympanic membrane. (**D**) In cholesteatoma, the location of the bony erosion was spotted in Grad-CAM.

**Figure 4:**
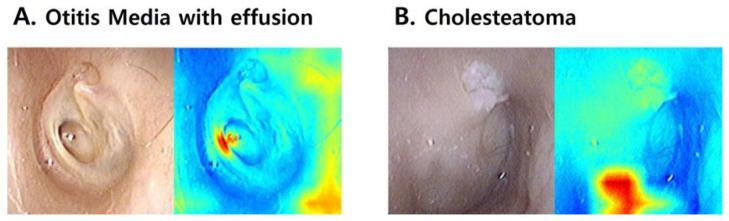
Gradient-weighted Class Activation Mapping (Grad-CAM) of the incorrectly predicted tympanic membrane images. (**A**) Otitis media with effusion was misdiagnosed as chronic otitis media because the machine learning model focused on the retracted part of the tympanic membrane and considered it as a perforation. (**B**) The machine learning model predicted a normal tympanic membrane by focusing on the pars tensa area of the tympanic membrane. The heat map image failed to highlight the small attic retraction and cholesteatoma materials.

Figure 3 shows the comparison between tympanic membrane images and Grad-CAM results according to diagnosis. The machine learning network was mainly interested in the tympanic membrane in the classification of normal and OME images. In the case of COM, the network focused on the perforated area and margin of the tympanic membrane. Cholesteatoma was highlighted in the area of external auditory canal destruction and the retraction pocket. The Grad-CAM of the incorrectly predicted images is described in Figure 4. OME was incorrectly determined as chronic otitis media because the machine learning network was confused in distinguishing the central retraction and perforation margin. In attic cholesteatoma, the network did not identify a small retraction pocket and assigned a diagnosis of normal tympanic membrane.

### 3.5. Performance of the Machine Learning Model for the Representative Data Set

**Figure 5:**
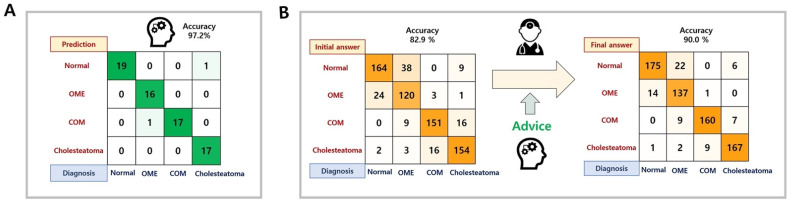
The confusion matrix for the diagnostic results for the machine learning network and resident physicians. (**A**) Prediction accuracy of the machine learning network in the representative cases, (**B**) The diagnostic performance change of ten resident physicians with the assistance of the machine learning network.

Figure 5a demonstrates the classification results of the machine learning network in 71 representative cases. The diagnostic accuracy of the machine learning network was 97.2%; the network made errors in determining “otitis media with effusion” and “cholesteatoma”.

### 3.6. Assistive Role of the Machine Learning Model for Determining Tympanic Membrane Status

**Figure 6:**
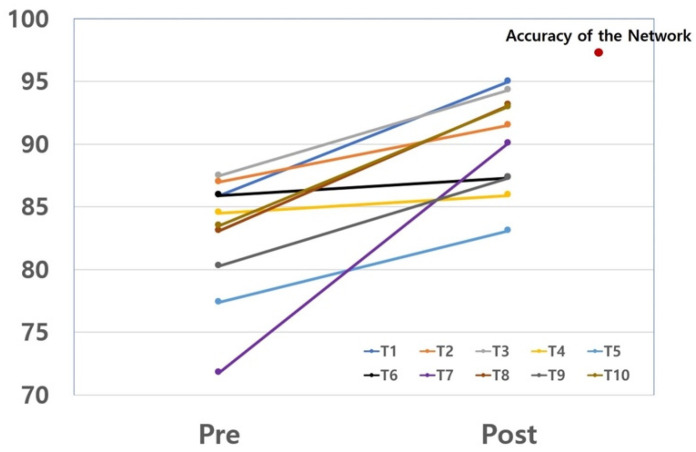
The diagnostic accuracy for middle ear diseases was increased in resident physicians after consultation with the machine learning model.

Of the 71 tympanic membrane images, initial diagnoses were correct in an average of 82.9% (77.4–87.5%) of cases. After machine learning assistance, the average correct diagnosis rate increased by 7.1% (1.4–18.3%) in Figure 5B. The individual enhancement of diagnostic accuracy was described in Figure 6. 

**Figure 7:**
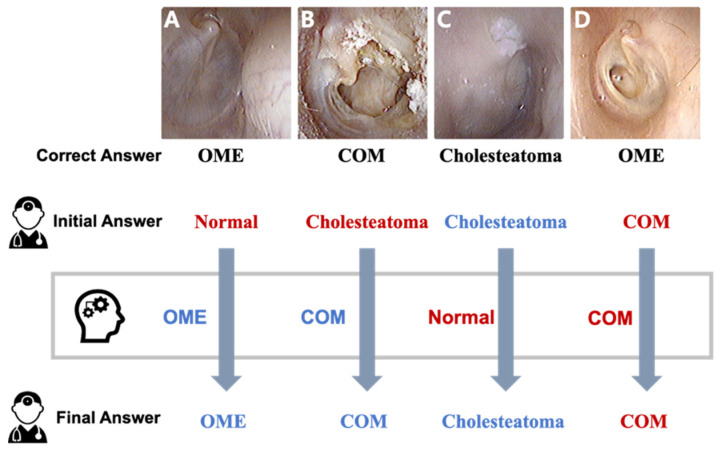
Representative cases in tympanic membrane images. (**A**,**B**) Many resident physicians changed to the correct answer with the advice of the machine learning network. (**C**) All resident physicians diagnosed cholesteatoma correctly despite wrong advice. (**D**) Resident physicians and the machine learning misdiagnosed OME as COM. OME: otitis media with effusion. COM: Chronic otitis media.

The most common error of resident physicians was in distinguishing between normal tympanic membrane and otitis media with effusion, and the second most common error was in differentiating between cholesteatoma and chronic otitis media. Figure 7 shows the representative tympanic membrane images by showing how physicians respond to the machine learning diagnosis. Many doctors had reviewed their first answers with the advice of the machine learning network and corrected the answers accordingly (Figure 7A,B). However, when doctors were convinced of the correct answer, they did not respond to the wrong advice from the network (Figure 7C), and there was a case both of them failed to diagnose correctly (Figure 7D).

## 4. Discussion

In this study, we developed a machine learning network to diagnose subtypes of otitis media and assessed the network’s ability to assist physicians in determining diagnoses from tympanic membrane images. The findings of this study were: (1) design of a diagnostic algorithm for otitis media, (2) a 97.2% diagnostic accuracy of the created network for representative cases, and (3) enhancement of the diagnostic accuracy of otolaryngology residents from 82.9% to 90.0% with algorithm assistance.

To the best of our knowledge, the present study is the first to assess the assistive or educational application of a machine learning network in a teaching hospital setting. The results are expected to contribute to the expansion of the application of machine learning-based diagnostic technology.

Otitis media refers to an inflammatory disorder in the middle ear cavity and is one of the most prevalent ear diseases in young children. Otitis media sufferers frequently seek medical attention, and the condition frequently results in the prescription of antibiotics and surgery [14,15]. Otitis media has a diverse disease spectrum with a characteristic tympanic membrane status. Otitis media with effusion is characterized by the presence of effusion behind the intact tympanic membrane, causing conductive hearing loss. Tympanic membrane perforation is the hallmark of chronic otitis media; presence of otorrhea refers to suppurative otitis media. Cholesteatoma is defined as keratinizing squamous epithelium having destructive and expanding growth leading to bony destruction [16]. Endoscopic images of cholesteatoma usually show attic destruction, granulation, and cholesteatoma debris.

In several studies, machine learning networks have been designed and applied to the classification of tympanic membranes in different settings. A previous study used Google’s Inception-V3 convolutional neural network architecture to detect tympanic membrane perforations in 233 tympanic membrane images [9]. The researchers reported an overall accuracy of 76.0% and suggested the possible application of a machine learning algorithm in the diagnosis of middle ear disease [9]. In addition, the computer-aided diagnosis of external and middle ear conditions was proposed. This system used a machine learning algorithm and support vector machine (SVM), k-nearest neighbor (k-NN), and decision trees with a 720-case training set. The group differentiated ear wax plug, myringosclerosis, chronic otitis media, and normal tympanic membrane with an accuracy of 93.8% [17]. Another study proposed a diagnostic system based on deep convolutional neural networks that achieved an average accuracy of 93.6%. These researchers classified six categories of ear conditions including attic retraction, tympanic perforation, myringitis, otitis externa, tumor, and normal tympanic membrane [8]. Since the diagnostic performance dropped to 80% with a decreased number of tympanic membrane images used for training, practical applications could be limited [8]. In addition, previous studies did not include a classification for otitis media with effusion into the tympanic membrane, a condition that is in practice difficult to differentiate from normal tympanic membrane [8,17]. The present study included an otitis media in the tympanic membrane classification, and the machine learning network successfully and accurately diagnosed otitis media with effusion. This was the most common misdiagnosis for all resident physicians (Figure 5B).

To increase the accuracy of the diagnostic network, we employed the attention mechanism, which is known to function well in image classification tasks by increasing the representation power. Specifically, we introduced a novel shuffle attention module that generates efficient 3D attention maps from the given input feature map. Moreover, we compared ResNet variants and evaluated their performance for our diagnosis task. As a result, ResNet18 with the proposed shuffle attention module showed the most accurate diagnostic performance among the ResNet variants. To enhance the exploitability of the machine learning network, we adopted Grad-CAM to demonstrate the activation level of the area contributing to the diagnosis. Consequently, the chosen diagnostic network produces reasonable predictions by focusing on the characteristic image findings in each middle ear disease.

The devised network showed a robust but imperfect accuracy in predicting tympanic membrane diseases. The network made a wrong diagnosis for two atypical cases of otitis media. Figure 4A demonstrates an OME case that was diagnosed incorrectly by the machine learning network and most resident physicians. All confused the thin, transparent, retracted part of the membrane as having a perforation. In Figure 4B, the machine learning network did not focus on small attic destruction and misdiagnosed the cholesteatoma case, while all resident physicians correctly diagnosed the attic cholesteatoma. Due to the lack of similar cases in the training data, it seems that these unusual conditions were not sufficiently learned by the algorithm. On the other hand, resident physicians frequently confused normal tympanic membranes with OME. However, the machine learning network always successfully differentiated normal tympanic membranes from OME, which is compatible with other previous reports showing the robust performance of machine learning networks in diagnosing OME in pediatric populations [18,19]. These complementary relationships between humans and machine learning networks suggest the future application of AI technology in medical practice.

This study assessed the educational or assistive role of a machine learning network in the diagnosis of middle ear disease via tympanic membrane images. In the experiment, resident physicians were not informed of the diagnostic accuracy of the machine learning network, which exceeded 97%, so we wanted to encourage them to reconsider the inconsistent cases instead of simply following the advised answers. After the initial diagnosis, the residents performed the second session receiving the advice of the machine learning network. On average, each physician failed to diagnose 12.4 of 71 images correctly at the first attempt (Figure 5). After advice from the network, 41% of the initial wrong diagnoses were corrected. Interestingly, the proportion of diagnosis changing varied from 10% to 65% and was not related to the grade of residency (Figure 6). We identified an assistive or educational role of the machine learning network in the assessment of tympanic membrane images.

Although the created network showed an excellent performance, the present study has several limitations. The present network successfully diagnosed representative middle ear disorders, but many other types of disease have not been addressed. We could not integrate diseases with a relatively low prevalence or those that were difficult to register with tympanic membrane images in a tertiary hospital. In the future, various ear diseases (for example, acute otitis media), various types of chronic middle ear conditions, and external auditory canal malignancy should be included to enhance the applicability of machine learning networks in actual practice. In addition, the tympanic membrane images used in this study were taken and preprocessed at a single institution. For universal use and to further improve performance with larger data, external validation processes with images from other institutions will be required, using diverse imaging acquisition methods and instruments.

## 5. Conclusions

The present study developed a high-performing machine learning network to diagnose middle ear diseases and identified the assistive role of a machine learning network for doctors in otolaryngology training.

## Figures and Tables

**Table 1:** Comparison of the diagnostic accuracy between the current network (ResNet18 + shuffle) and other available machine learning networks.

	ResNet18 + Shuffle	ResNet 18	ResNet18 + CBAM	ResNet50	ResNet50 + CBAM
Accuracy	97.183	94.366	92.958	94.366	91.549
Parameters	13 M	11 M	11 M	23 M	26 M

**Table 2:** Five-fold validation of the accuracy of the current network.

K-Fold	K-1	K-2	K-3	K-4	K-5	Average
Number	473	473	473	473	473	
Validation (accuracy, %)	93.446	93.446	92.389	93.658	95.56	93.69 ± 1.152

## Data Availability

The data presented in this study are available on request from the corresponding author.
